# Selective interaction and its effect on collective motion

**DOI:** 10.1038/s41598-022-12525-6

**Published:** 2022-05-21

**Authors:** Zhicheng Zheng, Xiaokang Lei, Xingguang Peng

**Affiliations:** 1 School of Marine Science and Technology, Northwestern Polytechnical University, Xi’an, 710072 People’s Republic of China; 2 School of Information and Control Engineering, Xi’an University of Architecture and Technology, Xi’an, 710055 People’s Republic of China

**Keywords:** Statistical physics, thermodynamics and nonlinear dynamics, Engineering

## Abstract

Plenty of empirical evidence on biological swarms reveal that interaction between individuals is selective. Each individual’s neighbor is selected based on one or more featured factors. Based on the self-propelled model, we develop a general probability neighbor selection framework to study the effect of four typical featured factors (i.e., distance, bearing, orientation change and bearing change). In this work, two common cases are involved to comprehensively analyze the impact of the four featured factors on the collective motion. One is the flocking, the other is the responsivity to stimulus. The impact of different selection strengths of the featured factors on both cases are investigated. The effect of noise on flocking and different stimulus intensities on responsivity to stimulus are analyzed. This study allows us to get the insight of selective interaction and suggests the potential solution to overcome the trade-off between flocking and responsivity quality.

## Introduction

Collective motion is a common phenomenon in nature, such as the colony of bacterial clusters^1^, school of fish^2^, flock of birds^3^ and active granular media^4^. Many efforts have been devoted to uncovering the mechanism of collective motion. Reynolds first proposed an approach to reproducing the flock of birds based on three simple rules (i.e., separation, alignment and cohesion), namely the Boid model^5^. Vicsek et al.^6^ proposed the simplest self-propelled model to investigate the phase transition in the non-equilibrium system and reproduced the ordered movement by velocity alignment. Aldana et al.^7^ described the original Vicsek model as the network system and found that the way that noise introduced into the system determines the order of phase transition in the self-propelled model. Chaté et al.^8^ studied the Vicsek model with cohesion and formed the liquid–gas phase transitions. Couzin et al.^9^ assumed the interaction between individuals is divided into three non-overlapping behavioral zones (i.e., zone of repulsion, orientation and attraction) and produced several complex collective behaviors by adjusting the width of each zone.

In recent years, plenty of empirical evidence has revealed selective interaction phenomena in animal groups^10–13^. Instead of considering all sensible neighbors, an individual selects its neighbors to react according to one or more featured factors. Such featured factors could be transient or dynamic determined by the relative motion of the focal individual. The transient featured factors are the stationary states of neighbors at a moment, such as the distance^5,6,9,14^ and bearing^15–17^. On the other hand, the dynamic featured factors are usually coupled with the time, which suggests that neighbor selection depends on the change of the neighbors’ states within a period, e.g., orientation change^18–20^ and bearing change^21,22^. Although different featured factors have been observed, there is no agreement on how to use these factors when modeling a desired collective motion. Comprehensive analysis is necessary to obtain an insight view about selective interaction.

To this end, in this paper we firstly developed a general probability framework of neighbor selection to study the effect of four typical featured factors (i.e., distance, bearing, orientation change and bearing change) on the collective motion based on the self-propelled model. Then, we considered two common cases of collective motion, i.e., the flocking and responsivity to stimulus when comprehensively analyzing the effects of featured factors. Specifically, the flocking suggests that individuals form the highly parallel-group^9^ from a disordered state. For the responsivity to stimulus, the stimulus can be interpreted as a kind of environmental cue^23^, which triggers the collective turn of the group. Additionally, the effects of different selection strength (i.e., the probability of being selected) of featured factors were analyzed in both cases. Also, different intensities of noise and stimulus were introduced when considering flocking and responsivity to stimulus, respectively. Interestingly, we found that solely increasing the strength of featured factors is harmful to the flocking; meanwhile, dynamic featured factors (i.e., orientation change and bearing change) are more responsive to the stimulus.

The rest of this paper is organized as follows. In “Methods” section, the general probability framework of neighbor selection is introduced to model self-propelled particle swarms. In “Results” section, the effects of each featured factor are analyzed from the perspective of selective interaction. The conclusions and further discussion are summarized in “Discussion” section.

## Methods

Here we consider a group composed of *N* individuals moving in the continuous two-dimensional *X*-*Y* plane without boundary. The position of the *i*-th individual changes according to

1 $$\begin{aligned} \dot{{{\mathbf {x}_\mathbf {i}}}} = \mathbf {v_i}, \end{aligned}$$

where $\mathbf {v_i}$ is the velocity of the *i*-th individual.

Each individual is self-propelled with a preferred speed $v_0$ and interacts with neighbors under random noise $\mathbf {\eta }$ (uniform distribution). The individual’s sensing radius is an infinite range, which suggested that all individuals could be the neighbor of individual *i*. The velocity of the *i*-th individual can be calculated as follows:

2 $$\begin{aligned} \dot{\mathbf {v}_\mathbf {i}} = \gamma _0 \left( 1 - \frac{\mathbf {{ v_i^2}}}{v_0^2}\right) \mathbf { { v_i}} + \mathbf {{ F_i}} + {\varvec{\eta }}_\mathbf {i}, \end{aligned}$$

where $\gamma _0$ is the relaxation rate, which characterizes the self-propelled force. An individual will reach the preferred velocity $v_0$ faster with a larger $\gamma _0$ and vice versa. The second term $\mathbf {{ F_i}}$ accounts for the interactions between individuals. For simplicity, we assume that $\mathbf {{\ F_i}}$ only depends on velocity alignment. Thus, ${\mathbf {F}_\mathbf {i}}$ is defined as $\mathbf {{ F_i}} = K(\mathbf {{v_d}} - \mathbf {{v_i}})$. *K* represents the strength of alignment and $\mathbf {{v_d}}$ is the velocity with which $\mathbf {{v_i}}$ should align.

**Figure 1:**
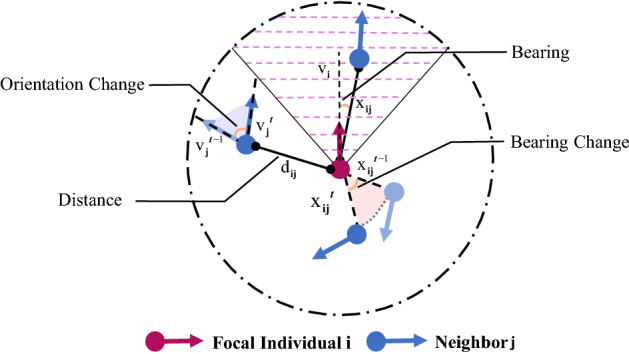
An illustration of the featured factors. Pink striped area represents the frontal preference of the focal individual *i*. ${\mathrm{d}_\mathrm{ij}} = ||\mathbf {x_i} - \mathbf { x_j}||$ is the distance between individuals *i* and *j* (Eq. (3)); $\mathbf {x_{ij}} = \mathbf {x_{j}} - \mathbf { x_{i}}$ is the relative position of *j* with respect to *i* (Eq. (4)); $\mathbf {v_{j}}^{t}, \mathbf { v_{j}}^{t-1}$ are the velocity vector of individual *j* at time *t* and $t-1$ in Eq. (5); $\mathbf { x_{ij}}^t, \mathbf { x_{ij}}^{t-1}$ are the position vector at time *t* and $t-1$ defined in Eq. (6), respectively.

Characterizing the neighbor selection according to different featured factors in a general way is the main part of our modeling framework. Inspired by Bode et al.^24^, we develop a probability framework. For a given individual *j*, the probability of being selected by the *i*-th individual obeys the following five selection models. Figure 1 shows the geometry of each featured factor.

### Random selection model (RAND-S model)

In the RAND-S model, the focal individual selects one neighbor randomly (uniform distribution) at each time step. This model is actually equal to the Vicsek model (all sensible neighbors’ states are averaged) if we consider time accumulation. In this paper, we use the RAND-S model as the baseline.

### Distance-based selection model (DIST-S model)

In the DIST-S model, the focal individual selects one neighbor at each time step according to the relative distances between the focal and its neighbors. The distance-based selection rule was first proposed by Reynolds^5^ and has been widely used to investigate the interaction network and the emergence of collective motion^6,9,14,25^. The probability of being selected in the DIST-S model is given as follows:

3 $$\begin{aligned} p_{ij}^{\mathrm{dist}} \sim \left( \frac{1}{||\mathbf {x_i} - \mathbf {x_j}||}\right) ^{c_d}, \end{aligned}$$

where $\mathbf {x_i}$ and $\mathbf {x_j}$ are the position of individual *i* and *j*, respectively. $c_d \ge 0$ is a parameter of tuning selection strength on the nearest neighbor. When the $c_d$ is small, individuals prefer the distant neighbors. On the contrary, with a large $c_d$, individuals tend to choose adjacent neighbors.

### Bearing-based selection model (BEAR-S model)

In the BEAR-S model, the focal individual selects on neighbor at each time step according to the bearing between the focal and its neighbors. The BEAR-S model is inspired by the evidence of anisotropic sensory behavior in some animal species, which suggests that neighbors’ bearing has an influence on the neighbor selection. Especially, the anisotropy of frontal preference has been found to have significant effect on the collective motion. For example, Pita et al.^15^ found that both the golden shiner and zebrafish have an acute vision in the fronto-dorsal region, which is in favor of group coherence. Lukeman et al.^17^ found that the interaction of surf scoters is within the frontal sector of $\pm \, 30^{\circ }$, which facilitates the collision avoidance in the group. The corresponding probability $p_{ij}^{\mathrm{bear}}$ is described as follows:

4 $$\begin{aligned} p_{ij}^{\mathrm{bear}} \sim \left( \frac{1}{\angle (\mathbf {v_i}, \mathbf { x_{ij}})}\right) ^{c_b}, \end{aligned}$$

where $\mathbf {v_i}$ represents the velocity (unit vector) of individual *i*, and $\mathbf {x_{ij}}$ is the unit position vector directed from *i* to *j*. $\angle (\cdot ,\cdot )$ represents the angle between two unit vectors. The strength of attention in front can be adjusted by $c_b \ge 0$. For a large strength $c_b$, the focal individual tends to choose the neighbor that appeared straight ahead.

### Dynamic-orientation selection model (DYO-S model)

Inspired by the result^18^ that the selection on neighbors is related to the orientation change of individuals rather than the distance in shoals of rummy-nose tetra species (Hemigrammus rhodostomus). Thus, we consider the orientation change within a period as a featured factor. For simplicity, we consider the time period as a single preceding step. The probability of DYO-S model is expressed as follows:

5 $$\begin{aligned} p_{ij}^{\mathrm{dyo}} \sim \left( \frac{\angle (\mathbf {v_{j}}^{t}, \mathbf {v_{j}}^{t - 1})}{\Delta t}\right) ^{c_o}, \end{aligned}$$

where $\Delta t$ is the step time interval in simulations. The $\mathbf {v_{j}}^{t}$ and $\mathbf {v_{j}}^{t - 1}$ are the velocity of individual *j* at time *t* and $t - 1$. $c_o \ge 0$ is used to adjust the strength of selection. For a large $c_o$, the focal individual tends to select the neighbor with the large orientation change.

### Dynamic-bearing selection model (DYB-S model)

Much empirical research has revealed that the animal could be sensitive to the bearing change of their neighbors through the visual pathway^21,22^. The change in bearing is the manifestation of the apparent movement, which conveys information that can directly influence the individuals’ movement. To be general, we assume that the time period of the bearing change is the same as that in the DYO-S model (i.e., one preceding step). The probability $p_{ij}^{\mathrm{dyb}}$ of selecting individual *j* can be described as follows:

6 $$\begin{aligned} p_{ij}^{\mathrm{dyb}} \sim \left( \frac{\angle (\mathbf {x_{ij}}^t, \mathbf {x_{ij}}^{t - 1})}{\Delta t}\right) ^{c_m}, \end{aligned}$$

where $\mathbf {x_{ij}}^t$ and $\mathbf {x_{ij}}^{t - 1}$ are the position vector directed from *i* to *j* at time step *t* and $t - 1$, respectively. $c_m$ is used to adjust the selection strength from small to large. When the $c_m$ is large, the focal individual tends to choose the neighbor with large bearing change.

To emphasize on the effect of the featured factors, we assumed that individuals only select one neighbor at each time step. Particularly, our framework includes the tunable parameter that controls the selection strength of the featured factors, which is able to select that specific neighbor from randomness to exclusiveness. In brief, our selection models comprise three steps.

1. Calculation on the cumulative distribution function (CDF) of being selected based on a certain featured factor (Eqs. (3)–(6)),
2. Choose one neighbor by sampling from the calculated CDF,
3. Update the position and velocity of each individual using Eqs. (1) and (2).

## Results

To investigate the property of each selection model, two cases are taken into account: flocking and responsivity to stimulus.

### Flocking

It is common for animal groups to take off at the same time and form the ordered movement spontaneously, which is crucial to maximizing the chance of survival^26,27^. In the flocking case, we measure the ability to form the ordered movement from the random initial state. Here, we consider two experimental parameters: the intensity of noise ${\eta }$ and the selection strength. The selection strength of each model is termed as follows: $c_d$ (DIST-S model), $c_b$ (BEAR-S model), $c_o$ (DYO-S model) and $c_m$ (DYB-S model).

Two metrics are used to evaluate the simulation results. First, the polarization $\phi$^6^ is used to evaluate the degree of consensus, which represents the flocking quality. The polarization $\phi$ is defined as follows:

7 $$\begin{aligned} \phi = \frac{1}{N}\left\| \sum _{i=1}^{N} \frac{\mathbf { v_i}}{\Vert \mathbf {v_i} \Vert }\right\| , \end{aligned}$$

here $\phi \in [0,1]$. When the group moves along with same direction, $\phi = 1$. Otherwise, if individuals diffuse with different directions, $\phi \approx 0$. Second, we evaluate the flocking efficiency using convergence time $T_{op}$^28^ which is the time consumption before the polarization $\phi$ first reaches the given threshold. The definition of $T_{op}$ is given as follows:

8 $$\begin{aligned} T_{op} = \mathop {min}\limits _{\phi> \varepsilon _{flock}} t, \quad t > 0, \end{aligned}$$

$\varepsilon _{flock}$ is the threshold of the ordered movement ($\varepsilon _{flock} = 0.95$ in this paper).

### Responsivity to stimulus

For biological swarm, the group constantly changes the moving direction to avoid the attack from predators. The responsivity to such sudden turning represents the effectiveness of information transfer within group^29^. To investigate the responsivity of featured factors, we consider the directional information as the stimulus. As suggested in the previous study^30^, a predefined number (i.e., ${\mathrm{IF}_\mathrm{num}}$) of informed individuals are randomly chosen from the group and abruptly turn with pi/2 respect to the current average moving direction. Moreover, we introduce stimulus intensity (i.e., the number of informed individual $\mathrm{IF}_\mathrm{num}$) and selection strength to conduct insightful analysis. Here, we use response accuracy $\delta _{group}$ and response time $T_{turn}$ to evaluate the quality and efficiency of the responsivity to stimulus, respectively. The response accuracy $\delta _{group}$ is defined as follows:

9 $$\begin{aligned} \delta _{group} = \frac{A - A_{no}}{1 - A_{no}}, \end{aligned}$$

where $A = \frac{1}{N} \sum _{i = 1}^{N} \frac{1 + \mathbf {v_i} \cdot \mathbf {h_s}}{2}$ is the average difference between the stimulus direction $\mathbf {h_s}$ with the velocity of each individual $\mathbf {v_i}$. $\mathbf {h_s}$ is orthogonal to the group’s moving direction. $A_{no}$ represents the degree of consensus between the group’s moving direction (averaged direction of all individuals) and the stimulus direction $\mathbf {h_s}$ at one preceding step of the informed individual’s turning moment, which is defined as $A_{no} = \frac{(1 + \mathbf {v_{no}} \cdot \mathbf {h_s})}{2}$. $\mathbf {v_{no}}$ is the group’s moving direction before the abruptly turning of the informed individual.

The time cost $T_{turn}$ during the response to stimulus is given as follows:

10 $$\begin{aligned} T_{turn} = \mathop {min}\limits _{\delta _{group}> \varepsilon _{turn}} t, \quad t > t_{group}, \end{aligned}$$

here $\varepsilon _{turn}$ is the threshold if the group completes the response. $t_{group}$ is the turning moment of the informed individual. We set $\varepsilon _{turn} = 0.9$ and $t_{group} = 10$ in the following simulations.

Simulations are run with the following parameters: $N = 100$, $\Delta t = 0.02$, $v_0 = 5$, $\gamma _0 = 5$ and $K = 3$. The number of informed individual ${\mathrm{IF}}_\mathrm{num}$ is no more than 5% of total number ($\mathrm{IF}_\mathrm{num} \in [1,5]$). All simulations are executed for 800 steps. Unless otherwise specified, any selection model discussed in this paper is the same as the RAND-S model if the selection strength is equal to zero.

**Figure 2:**
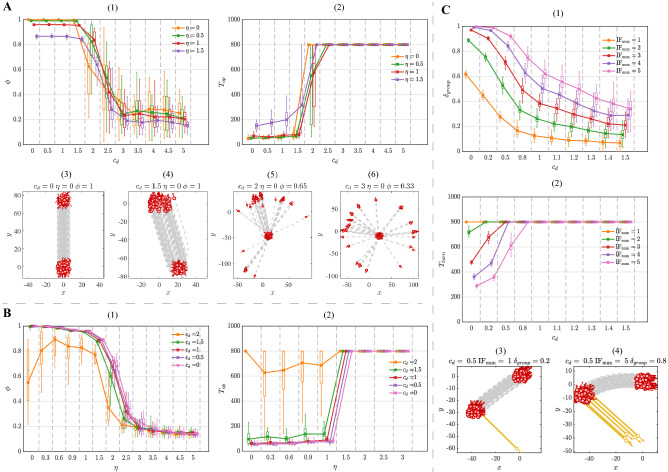
(**A**) The property of flocking with the evolution of $c_d$ in the DIST-S model. (**A**-(1)) The polarization $\phi$ as a function of $c_d$. (**A**)-(2)) The convergence time $T_{op}$ as a function of $c_d$. (**A**-(3–6)) The trajectories of flocking with different $c_d$. (**B**) The property of flocking with the evolution of noise $\mathrm \eta$ in the DIST-S model. (**B**-(1)) The polarization $\phi$ as a function of noise $\mathrm \eta$. (**B**-(2)) The convergence time $T_{op}$ as a function of noise $\mathrm \eta$. (**C**) The property of responsivity to stimulus with the evolution of $c_d$ in the DIST-S model. (**C**-(1)) The response accuracy $\delta _{group}$ as a function of $c_d$. (**C**-(2)) The response time $T_{turn}$ as a function of $c_d$. (**C**-(3–4)) The trajectories of response to stimulus with different $c_d$. $T_{op} = 800$ or $T_{turn} = 800$ indicate that the group is unable to finish the corresponding case within the total simulation time. Each data point represents the average of 50 simulation runs with the boxplot. Boxplots in the block divided by the dotted line belong to the same data point but are shifted at different positions on the x-axis to be more distinguishable.

### Distance-based selection model

**Figure 3:**
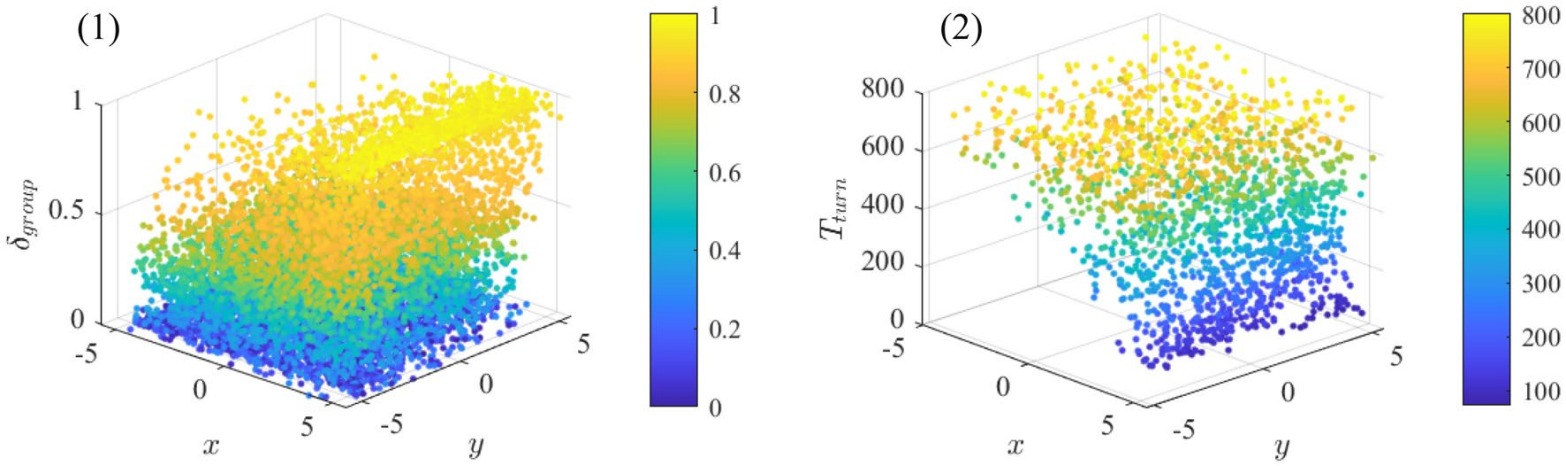
The responsivity of the BEAR-S model with the single informed individual (${\mathrm{IF}_{\mathrm{num}} = 1}$, $c_b = 20$). (1) Response accuracy $\delta _{group}$. (2) The time cost of response $T_{turn}$ (simulations of the uncompleted response is not drawn).

**Figure 4:**
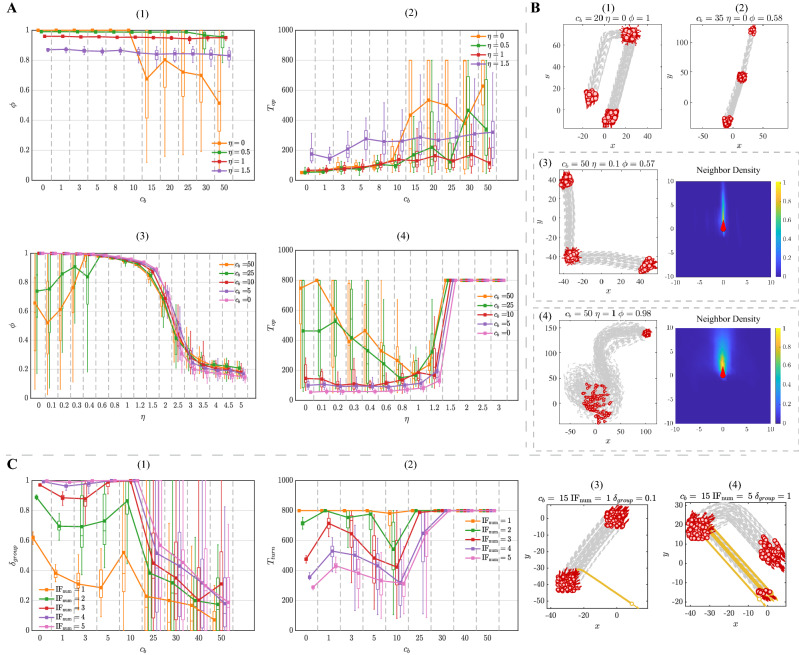
(**A**) The property of flocking as the evolution of $c_b$ and $\mathrm \eta$ in the BEAR-S model. (**A**-(1)) The polarization $\phi$ as a function of $c_b$. (**A**-(2)) The convergence time $T_{op}$ as a function of $c_b$. (**A**-(3)) The polarization $\phi$ as a function of noise $\mathrm \eta$. (**A**-(4)) The convergence time $T_{op}$ as a function of noise $\mathrm \eta$. (**B**) The trajectories of flocking with different $c_b$. (**C**) The property of responsivity to stimulus with the evolution of $c_b$ in the BEAR-S model. (**C**-(1)) The response accuracy $\delta _{group}$ as a function of $c_b$. (**C**-(2)) The response time $T_{turn}$ as a function of $c_b$. (**C**-(3–4)) The trajectories of response to stimulus with different $c_b$. $T_{op} = 800$ or $T_{turn} = 800$ indicate that the group is unable to finish the corresponding case within the total simulation time. Each data point represents the average of 50 simulation runs with the boxplot. Boxplots in the block divided by the dotted line belong to the same data point but are shifted at different positions on the x-axis to be more distinguishable.

**Figure 5:**
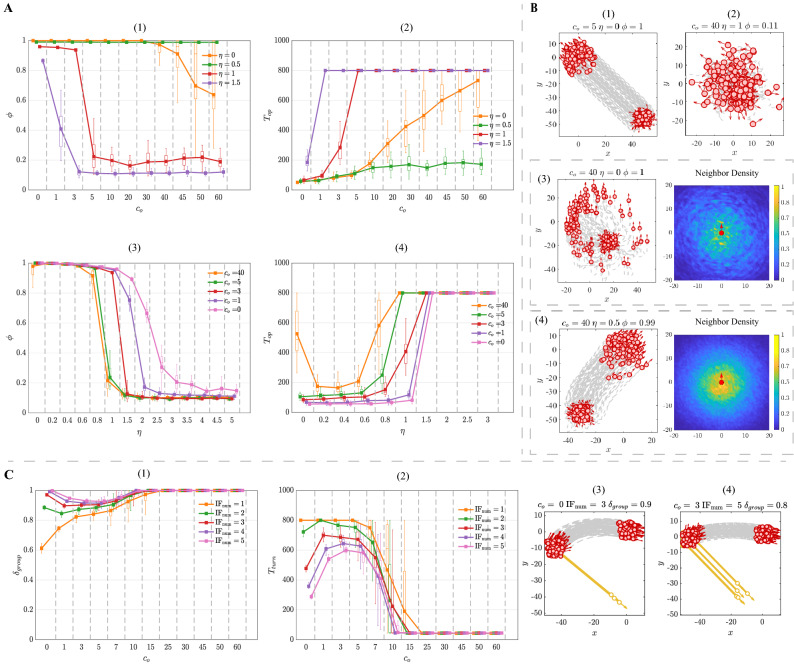
(**A**) The property of flocking as the evolution of $c_o$ and $\mathrm \eta$ in the DYO-S model. (**A**-(1)) The polarization $\phi$ as a function of $c_o$. (**A**-(2)) The convergence time $T_{op}$ as a function of $c_o$. (**A**-(3)) The polarization $\phi$ as a function of noise $\mathrm \eta$. (**A**-(4)) The convergence time $T_{op}$ as a function of noise $\mathrm \eta$. (**B**) The trajectories of flocking with different $c_o$. (**C**) The property of responsivity to stimulus with the evolution of $c_o$ in the DYO-S model. (**C**-(1)) The response accuracy $\delta _{group}$ as a function of $c_o$. (**C**-(2)) The response time $T_{turn}$ as a function of $c_o$. (**C**-(3–4)) The trajectories of response to stimulus with different $c_o$. $T_{op} = 800$ or $T_{turn} = 800$ indicate that the group is unable to finish the corresponding case within the total simulation time. Each data point represents the average of 50 simulation runs with the boxplot.Boxplots in the block divided by the dotted line belong to the same data point but are shifted at different positions on the x-axis to be more distinguishable.

**Figure 6:**
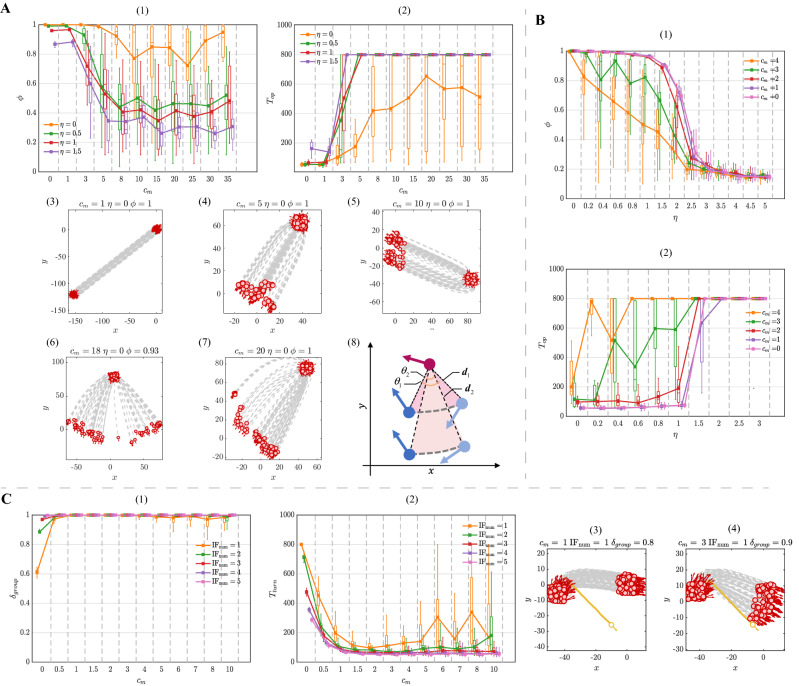
(**A**) The property of flocking as the evolution of $c_m$ in the DYB-S model. (**A**-(1)) The polarization $\phi$ as a function of $c_m$. (**A**-(2)) The convergence time $T_{op}$ as a function of $c_m$. (**A**-(3–6)) The trajectories of flocking with different $c_m$. (**A**-(3–7)) The illustration of bearing change of neighbor with different distance. When the distance $d_1 < d_2$, it is evident to find that $\theta _1 > \theta _2$ if the traveling distance is the same. (**B**) The property of flocking as the evolution of $\eta$ in the DYB-S model. (**B**-(1)) The polarization $\phi$ as a function of noise $\eta$. (**B**-(2)) The convergence time $T_{op}$ as a function of noise $\eta$. (**C**) The property of responsivity to stimulus with the evolution of $c_m$ in the DYB-S model. (**C**-(1)) The response accuracy $\delta _{group}$ as a function of $c_m$. (**C**-(2)) The response time $T_{turn}$ as a function of $c_m$. (**C**-(3–4)) The trajectories of response to stimulus with different $c_m$. $T_{op} = 800$ or $T_{turn} = 800$ indicate that the group is unable to finish the corresponding case within the total simulation time. Each data point represents the average of 50 simulation runs with the boxplot. Boxplots in the block divided by the dotted line belong to the same data point but are shifted at different positions on the x-axis to be more distinguishable.

As for the DIST-S model, the preference on the nearest neighbor weakens the ability to form the ordered movement. Under the non-noise condition, when the selection strength is small ($c_d < 1.5$), the polarization $\phi$ and convergence time $T_{op}$ keep around the optimal value (Fig. 2A-(1–2)), which suggests that individuals achieve the flocking efficiently (Fig. 2A-(4)). However, when the selection strength is large ($c_d > 1.5$), the group splits into a few subgroups with different sizes and moving directions (Fig. 2A-(3–5)), which implies the declination of flocking quality. Moreover, as the further increase of selection strength $c_d > 3$, individuals are scattered around and completely collapse (Fig. 2A-(6)). This is because the focal individual overlooks the other individuals and only aligns with the nearest neighbor, which leads to the pairwise interaction. As a result of that, the slight directional deviation between individuals is hard to eliminate in time, resulting in the transform of group formation from coherence to disperse and the decay of flocking quality.

Under the noise condition, it is inevitable that the polarization and flocking efficiency decline with the noise increases. As shown in Fig. 2B-(1–2), the movement of individuals is disrupted by large noise intensity ($\eta > 2$). When the noise intensity and selection strength is both small ($\eta < 1$ and $c_d < 1.5$), the group is able to form the ordered movement efficiently. However, when the selection strength $c_d > 1.5$, the polarization and flocking efficiency significantly reduce under the same noise intensity ($\eta < 1$). To sum up, the strong preference on the nearest neighbor has a negative impact on noise tolerance.

The responsivity to stimulus is promoted by the preference on the distant neighbor rather than the nearest neighbor. According to Fig. 2C-(1), when the stimulus intensity is weak ($\mathrm{IF}_\mathrm{num} = 1$), the response accuracy $\delta _{group}$ never exceeds 0.5, which means that the group is unable to response to the stimulus (Fig. 2C-(3)). Additionally, from Fig. 2C-(1–2), it can be seen that the response accuracy and efficiency show an apparent upward trend with the stimulus intensity increases, which implies that individuals require more stimulus information to make response. The trajectory of the response under a large stimulus intensity ($\mathrm{IF}_{\mathrm{num}} = 5$) is shown in Fig. 2C-(4). However, under the maximum stimulus intensity ($\mathrm{IF}_{\mathrm{num}} = 5$), the response accuracy and efficiency still decrease consistently as the increase of $c_d$. Basically, the reason for low responsivity is that individuals only focus on their nearest neighbors during the response process, which blocks the stimulus information transfer within the group. In other words, directional information is hard to efficiently spread in the DIST-S model.

### Bearing-based selection model

For the BEAR-S model, the frontal preference has a negative impact on forming ordered movement. In the non-noise situation, according to Fig. 4A-(1–2), when the frontal preference is weak ($c_b < 10$), individuals achieve the ordered movement efficiently. As the increase of $c_b$, the group tends to split into two or more clusters. These clusters are still likely to form the ordered movement when the frontal preference is at an intermediate level ($c_b \approx 20$), as shown in Fig. 4B-(1). When the selection strength is large ($c_b > 25$), the group splits into two clusters with totally different moving directions (Fig. 4B-(2)). Meanwhile, the polarization $\phi$ declines to 0.6, and the convergence time $T_{op}$ rises to 600 simulation steps. It arises from the fact that the frontal preference restrains the individuals’ interaction range. The neighbor in front directly affects the individual at the back. On the contrary, the back-end individual never has an influence on the neighbor in front. In other words, the interaction becomes regional and its topology switches slowly. This leads to the group dispersion and it is difficult for individuals to keep ordered.

In the noise situation, it is interesting that different strength of frontal preference leads to quite different decay tendencies. When the selection strength is small ($c_b < 10$), the polarization and flocking efficiency decline monotonically with the increase of noise intensity. Counterintuitively, under the strong frontal preference ($c_b > 10$), the BEAR-S model exhibits a non-monotonic decline tendency. Specifically, as shown in Fig. 4A-(3–4), when the noise intensity varies in $\eta \in [0, 1]$, the polarization $\phi$ takes an upward trend while the convergence time $T_{op}$ has a rapid decrease. This suggests that under this situation the flocking quality and efficiency have been promoted evidently. The polarization and flocking efficiency achieve the optimal value at $\eta \approx 0.8$ rather than that under the non-noise condition ($\eta = 0$). It is because that the involvement of noise makes it easier for individuals to interact with more neighbors. As shown in Fig. 4B-(3–4), the neighbor density under the noise $\eta = 0.1$ (Fig. 4B-(3)) is narrower than that under the large noise intensity $\eta = 1$ (Fig. 4B-(4)). The individuals’ interaction range is expanded to the larger level, which means that the flocking quality is promoted as the result of noise perturbation.

Frontal preference is negative for the stimulus transfer within the group. According to the Fig. 4C-(1–2), with the increase of $c_b$, the general trend in response accuracy $\delta _{group}$ decreases consistently while the response time $T_{turn}$ shows an apparent growth for any number of the informed individual. More evidence can be seen in Fig. 4C-(3), individuals with strong frontal preference barely respond to the stimulus. The group cannot follow the turning of the informed individuals until the stimulus intensity increasing to $\mathrm{IF}_{\mathrm{num}} = 5$ (Fig. 4C-(4)). Nevertheless, with the increase of $c_b$, the response accuracy and efficiency still decrease rapidly. It is due to that the strong frontal preference limits the individuals’ perception and breaks the stimulus propagation link within the group. As shown in the Fig. 4C-(1–2), the variance of response accuracy $\delta _{group}$ and response time $T_{turn}$ are both high. There is another interesting finding shown in Fig. 3, the informed individual in the front of the group does promote the responsivity to stimulus, but it is still unable to transfer the stimulus effectively on occasions. The reason might be that the frontal preference is highly related to the neighbors’ bearing. Thus, the group responsivity becomes sensitive to the the initial position of stimulus (i.e., the spatial distribution of the informed individual).

### Dynamic-orientation selection model

For the DYO-S model, the polarization is not sensitive to the increase of $c_o$, while the flocking efficiency declines significantly with large selection strength $c_o$. As shown in Fig. 5A-(1), in non-noise situation, when the selection strength $c_o$ becomes extremely large ($c_o \approx 60$), the polarization $\phi$ declines to 0.65. At the same time, the group is slightly dispersed and keeps the ordered formation (Fig. 5B-(1)). As for the flocking efficiency, as shown in Fig. 5A-(2), the convergence time $T_{op}$ rises consistently with the increase of $c_o$. The decrease of flocking efficiency is due to that the orientation change of each individual is lack of discrepancy. The switching of neighbors is driven by the stochastic factor in simulations. The effectiveness of neighbor selection is heavily reduced, which leads to the blind movement.

In the noise situation, the noise tolerance is impaired by the large selection strength $c_o$. According to Fig. 5A-(3), when the selection strength $c_o$ is small ($c_o < 1$), the group is able to keep the high polarization even with the noise intensity $\eta = 1.5$. However, with the increase of $c_o$, individuals become vulnerable to the noise. As for the polarization, the decline tendency shifts from a gradual decrease to a rapid drop as the approximately discontinuous transition manner. The critical noise intensity of the disorder–order transition is decayed from $\eta \approx 1.5$ ($c_o$ = 1) to $\eta \approx 0.8$ ($c_o$ = 40). The DYO-S model’s intrinsic mechanism (i.e., identification of the neighbor with a large orientation change) is responsible for the significant impairment of the noise tolerance. Individuals suffer from the false information (i.e., the wrong alignment direction) under the large selection strength $c_o$, which means that the group cannot form the ordered movement (Fig. 5B-(2)).

As for the flocking efficiency, as shown in Fig. 5A-(4), with the increase of noise, different selection strength $$c_o$$ results in the different decline tendencies of flocking efficiency. Specifically, when the selection strength is small ($$c_o < 1$$), it is reasonable to find that the convergence time $$ T_{op}$$ maintains an upward trend with the increase of noise intensity. Counterintuitively, with the large selection strength ($$c_o \approx 40$$), the effect of noise on the convergence time $$ T_{op}$$ shows a non-monotonic decline tendency. Interestingly, as shown in Fig. 5A-(4), there is an unexpected decrease in the convergence time $$T_{op}$$ with the noise intensity ($$\eta \in [0, 0.5]$$). Such promotion of flocking efficiency is mainly attributed to that the neighbor switch becomes more frequent under the noise condition, which implicitly expands the interaction range. As shown in Fig. 5B-(3–4), comparing with the condition of noise intensity $$\eta = 0.1$$, the interaction range is expanded by larger noise intensity $$\eta = 0.5$$.

The large selection strength $c_o$ improves the response accuracy and efficiency to the stimulus. As shown in Fig. 5C-(1–2), under the low stimulus intensity (${\mathrm{IF}_{\mathrm{num}} = 1}$) and also the increase of $c_o$, the response accuracy $\delta _{group}$ rises up fast while the response time $T_{turn}$ decreases significantly. Even if the selection strength is large ($c_o > 10$), the response accuracy $\delta _{group}$ and time cost $T_{turn}$ remain stable at their optimal values regardless of stimulus intensity changes. However, when the selection strength $c_o < 10$, the response accuracy and efficiency have two different trends depending on the different levels of stimulus intensity. On the one hand, when the stimulus intensity ${\mathrm{IF}_{\mathrm{num}} \le 2}$, the response accuracy and efficiency increase monotonically to the optimal value. On the other hand, with the large stimulus intensity (${\mathrm{IF}_{\mathrm{num}} \ge 3}$), the promotion of responsivity shows a non-monotonic tendency (i.e., first decreasing and then increasing). For example, when the stimulus intensity $\mathrm{IF}_{\mathrm{num}}$ is up to 3, it is sufficient for the group to respond under the influence of average interaction ($c_o = 0$), as shown in Fig. 5C-(3). Since the focal individual takes the limited focus on the neighbor with large orientation change ($c_o < 5$), the influence of average interaction is neutralized and leads to the reduction on responsivity to stimulus (Fig. 5C-(4)).

### Dynamic-bearing selection model

For the DYB-S model, the large selection strength $c_m$ leads to the decrease in polarization and has an unfavorable effect on the flocking efficiency. In the non-noise situation, as shown in Fig. 6A-(1), with the increase of $c_m$, the polarization falls gradually at $\phi \approx 0.8$. Meanwhile, as shown in Fig. 6A-(3–7), the group disperses laterally as a line-like formation but still forms the ordered movement. According to Fig. 6A-(2), the convergence time $T_{op}$ shows an evident upward trend. When the selection strength $c_m > 20$, the flocking efficiency reaches the lowest level ($T_{turn} \approx 500$ simulation steps). The reason for the decrease in flocking efficiency is that preference on neighbors is changed. Specifically, when the selection strength $c_m$ is large, the individuals’ preference on neighbors converts from the bearing change to the close proximity. Additionally, when traveling with the same distance, the bearing change is more noticeable for the closer individuals, as shown in Fig. 6A-(8). This conversion leads to the regional interaction between individuals. It is difficult for the group to keep coherent and achieve the ordered movement efficiently.

In the noise situation, the group that pays more attention to the neighbor with the large bearing change has less tolerance on the noise. From Fig. 6B-(1), we can see that the polarization declines from gradually to rapidly with the increase of $c_m$. When the selection strength $c_m$ is small ($c_m < 2$), individuals are able to keep the high polarization and flocking efficiency when the noise intensity $\eta < 1$ (Fig. 6B-(1–2)). When the selection strength $c_m$ is large ($c_m > 2$), polarization and flocking efficiency declines accordingly under the same noise intensity ($\eta < 1$). This is because that the noise affects the neighbors’ bearing perception and confuses individuals during the neighbor selection process.

The preference on the neighbor with the large bearing change allows the group to transfer the stimulus accurately and efficiently. According to the Fig. 6C-(1–2), even with the low stimulus intensity ($\mathrm{IF}_{\mathrm{num}} = 1$), the response accuracy $\delta _{group}$ increases rapidly and the response time $T_{turn}$ falls sharply as the increase of $c_m$. When the selection strength $c_m > 2$, the response accuracy $\delta _{group}$ and efficiency reaches their optimal value, respectively. Moreover, with the increase of stimulus intensity, the responsivity of the group keeps at the optimal level regardless of any selection strength $c_m$ ($c_m > 0$). The reason for such great responsivity is that the interaction topology that emerged in the group facilitates the stimulus transfer. In other words, the interaction topology based on bearing change can efficiently spread the directional information within the group.

## Discussion

In summary, we analyzed the effect of different featured factors on the collective motion based on the probability framework, as it is necessary to get the insight of the selective interaction.

For the flocking, in the non-noise situation, it is important to find that the increase of selection strength has a negative impact on the flocking for both transient and dynamic featured factors. In the noise situation, it is interesting that the proper noise intensity promotes the flocking quality in both the BEAR-S model and the DYO-S model.

On the other hand, the responsivity of different featured factors differs greatly. The information transfer by dynamic featured factors (i.e., orientation change, bearing change) is more efficient than that through the transient. Generally, the most significant difference between the dynamic and transient featured factors is the introduction of the change of neighbor’s state. The dynamic featured factors consider the neighbors’ state at not only a particular moment but also the collection of the neighbor’s state over a period. The dynamic featured factor represents the gradient of the stimulus. Comparing with the transient featured factors, the dynamic featured factors are intrinsically suitable to transfer the stimulus within the group.

According to the simulation results, we found that there is a trade-off between the flocking quality $\phi$ (i.e., persistence) and the responsivity quality $\delta _{group}$. Especially for the DYO-S and DYB-S model, the group is bound to choose either high flocking quality or high responsivity when they exhibit collective motion. Persistence and responsivity are two distinct perspectives of collective motion, which can not be achieved simultaneously. The persistent (coordinated) movement is achieved by ruling out deviations from group’s moving direction, which demands the overdamped interaction dynamics. However, the responsivity is obtained by spreading fast turns across the group, which requires the underdamped interaction dynamics^23,31^. In this work, our results suggest that there may be two possible ways to reconcile this trade-off in the view of selective interaction. For one thing, the combination of different featured factors could be an effective solution. As an instance, the DYB-S model incorporated with the proper frontal preference (i.e., BEAR-S model) is likely to promote the noise tolerance and remain the high responsivity to stimulus. For another thing, featured factors shift in accordance with different tasks. At the beginning of the flocking, it is reasonable to adopt the distance in the collective model for its simplicity. In addition, the neighbor’s bearing (i.e., the frontal preference) could be involved to improve the noise tolerance in the complex environment. Importantly, when individuals are under stimulus, the featured factors of change in orientation and bearing have potential to promote the responsivity of the group. Switching the featured factors in the collective model will make it easy to handle different situations.

In this paper, we only involve the single-neighbor interaction in our proposed framework. However, the number of selected neighbors is another significant part of the neighbor selection strategy. It has been shown that the emergent coordinated movement depends strongly on how many neighbors a focal individual can pay attention to in Refs.^32–34^. To be general, we also conduct some preliminary experiments on the interaction with multiple neighbors in the case of flocking. The main conclusions remain consistent with the experiments of single-neighbor interaction. The general trend of flocking quality $\phi$ and efficiency $T_{op}$ declines with the increase of selection strength for any number of selected neighbors. Interestingly, with the increase of the number of selected neighbors, the declination trend of flocking quality and efficiency becomes much smoother, which suggests that further analysis is necessary to have a better understanding on the effects of multi-neighbor interaction. Additionally, there is no agreement on the number of selected neighbors in the biological swarm, which might be different from animal species, e.g., the starling birds usually interact with 6–7 neighbors^16^, while the interaction in the fish group appears to be restricted to a rather low number of neighbors (one or two individuals)^14,18^. Therefore, we plan to comprehensively investigate the influence of the number of selected neighbors on a robust ordered motion in the future.

## Data Availability

The datasets generated and/or analysed during the current study are available from the corresponding author on reasonable request.
